# Lichen Planus Triggered by Dengue Infection: An Unusual Clinical Presentation

**DOI:** 10.1002/ccr3.70595

**Published:** 2025-07-06

**Authors:** Sunil Jaiswal, Shraddha Uprety, Pratichya Thapa, Prakriti Lamichhane

**Affiliations:** ^1^ Department of Dermatology Chitwan Medical College Bharatpur Nepal

**Keywords:** clinical presentation, dengue fever, dermoscopy, lichen planus

## Abstract

Systemic viral infections have been implicated as possible triggers for Lichen planus. An association of Lichen planus with dengue infection is exceptionally rare. We report a case of 38‐year‐old male who developed Lichen planus after the onset of dengue fever without any prior history or family history of Lichen planus. This case underscores the potential for dengue virus to act as a precipitating factor for Lichen planus, emphasizing the importance of identifying and managing the condition in clinical settings.

**Summary**

- Our observation highlights a rare but notable association between dengue infection and the onset of Lichen planus. As a clinician, one should remain vigilant for atypical clinical presentation especially in post‐viral states for timely recognition and appropriate management of the disease condition.

## 1 Introduction

Lichen planus is an autoimmune chronic inflammatory condition affecting skin, nails, hair, and mucous membranes [1]. The prevalence of Lichen planus is found to be 0.2%–1% worldwide [2]. Genetic and environmental factors, including infections and drugs, are the common etiological factors of Lichen planus. T lymphocytes play a major role in the etiopathogenesis of the disease, which causes destruction of the basal keratinocytes [3]. Patients usually present with itchy, bilaterally symmetrical, violaceous papules and plaques on the flexor surfaces of the extremities [4].

Here, we report a noteworthy case of Lichen planus with an unusual presentation in a 38‐year‐old man triggered by Dengue infection.

## 2 Case History and Examination

**FIGURE 1:**
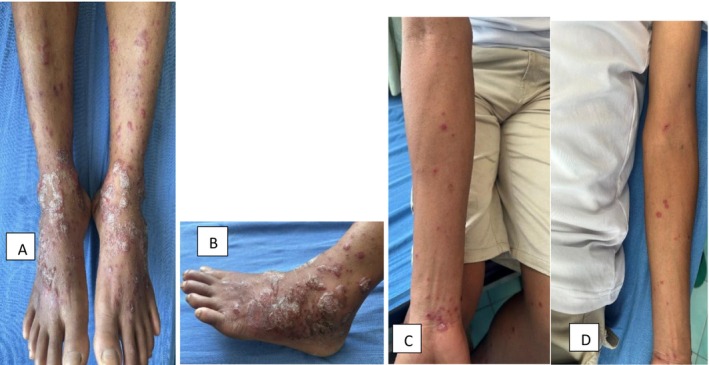
(A, B) Bullae and scaly plaques on the foot and distal leg. (C, D) Erythematous papules and crusted lesions over proximal legs, thighs, and forearm.

A 38‐year‐old male, army personnel presented to our OPD with bullae and scaly plaques over the foot and distal leg for the past 3 days, Figure 1A,B. He also had erythematous papules and crusted lesions over the proximal legs, thighs and forearm, Figure 1C,D. The lesions were associated with mild itching. He had been experiencing episodes of fever, malaise, and joint pain for the past 7 days, prior to the onset of the skin lesions.

The patient denied history of Lichen planus and any known co‐morbidities in the family. Investigations were sent to evaluate the cause of febrile illness. Complete Blood Count revealed low leucocyte count (3200/mm^3^) and low platelet count (50,000/mm^3^). Dengue serology showed positive for NS1 antigen and IgM antibody. Histopathological examination could not be done because of a significantly low platelet count.

## 3 Provisional Diagnosis

The provisional diagnosis considered was viral exanthematous rash.

## 4 Outcome and Follow‐Up

**FIGURE 2:**
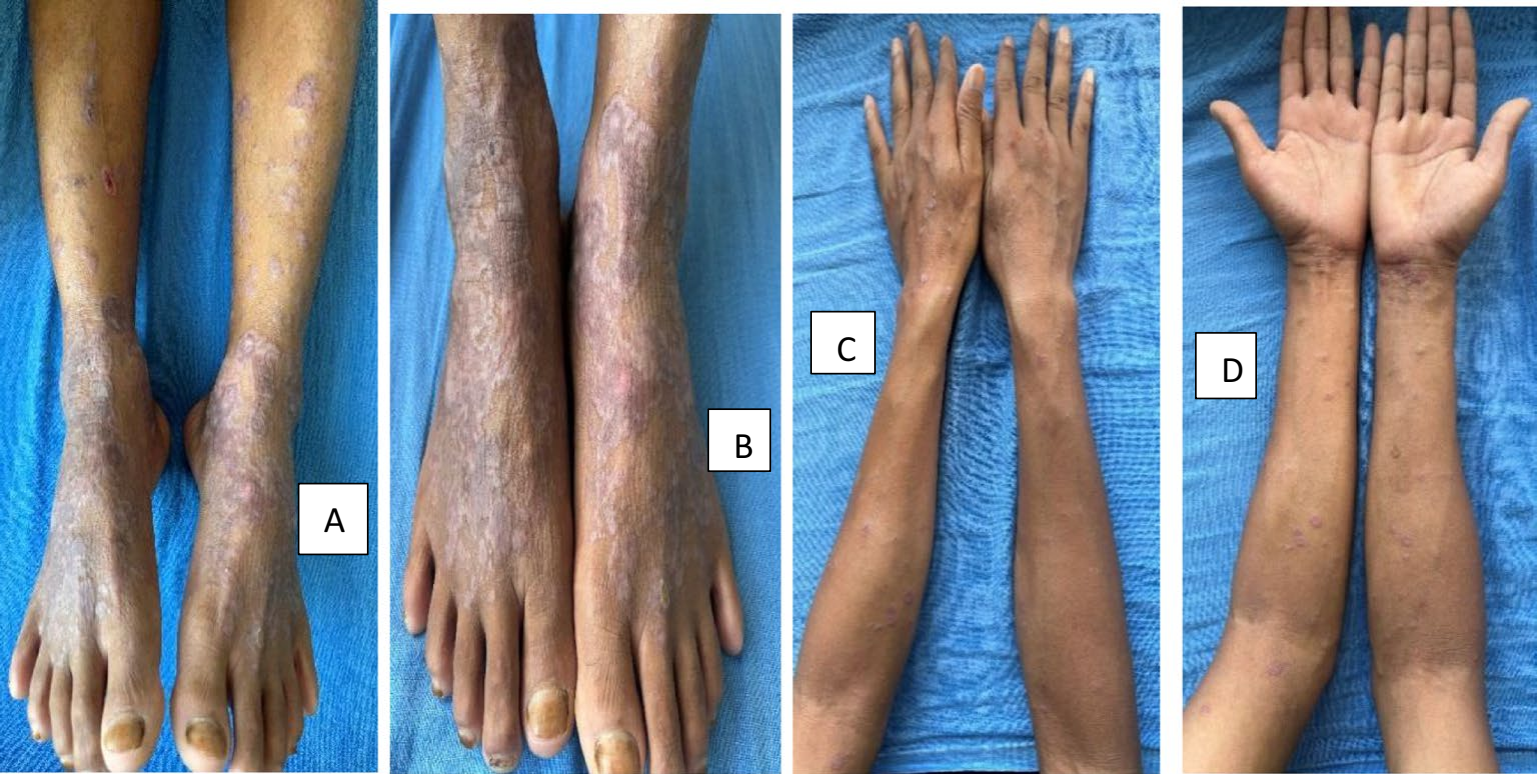
(A–D) Annular itchy violaceous plaques and flat‐topped violaceous papules over the previous lesions.

**FIGURE 3:**
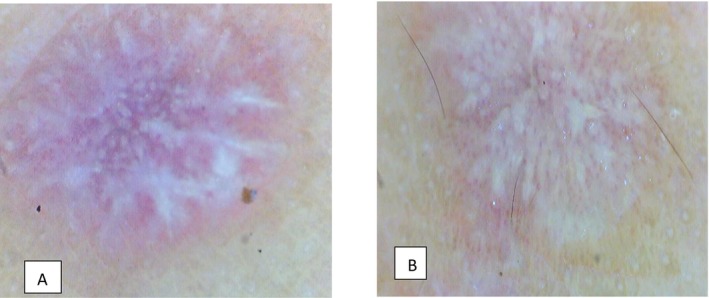
(A, B) Dermoscopy of violaceous lesions revealed Wickham's striae and pink globules.

**FIGURE 4:**
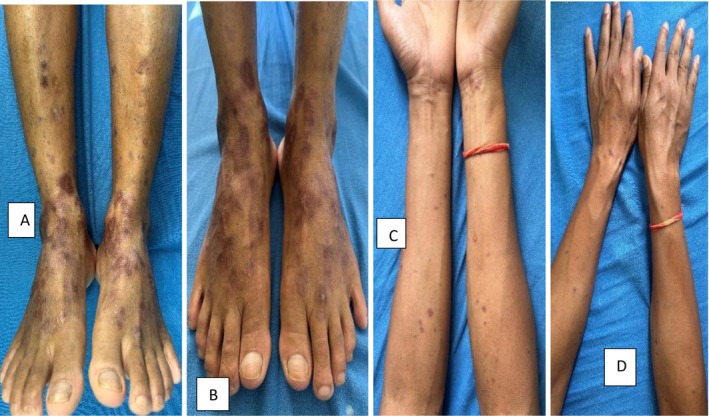
(A–D) Lesions of Lichen planus healing with hyperpigmentation after a course of steroid.

Patient was then admitted in the ICU for further management from Medicine Department with symptomatic management from Dermatology Department. Patient's condition improved and he was discharged after 10 days of hospital stay. After around 2 weeks of hospital admission, the initial lesions subsided and the patient developed itchy flat‐topped violaceous plaques and papules over the previous lesions [Figure 2A–D]. Dermoscopy of the violaceous lesions revealed Wickham's striae and pink globules [Figure 3A,B]. Hence, the patient was diagnosed with Lichen planus (LP) based on clinico‐dermoscopic evaluation. The patient was started on oral prednisolone (0.5 mg/kg/day). Significant improvement of the lesions was evident after 2 weeks of oral prednisolone. Oral prednisolone was tapered and the lesions resolved permanently after the patient was healed from Dengue fever [Figure 4A–D].

## 5 Discussion

The patient in our study had scaly erythematous plaques, papules, and few bullae at initial presentation which evolved into Lichen planus (LP) following the onset of Dengue fever. Dengue infection might have played an instrumental role in the variable presentation of the cutaneous disease.

Lichen planus (LP) is an autoimmune disease with various proposed etiologies. Systemic infection, metals, drugs, contact allergens, stress, and vaccination are considered to be potential triggers of Lichen planus. Systemic viral infections that are associated with the occurrence of LP include hepatitis C, members of the human herpes virus family, Varicella Zoster virus, human papillomavirus, Epstein Barr virus, and SARS‐COV2 [3].

The unusual presentation of Lichen planus as evidenced in our patient is quite rare and thus the mechanism of such an evolution is not exactly known. In our patient, LP developing over the previous scaly erythematous plaques and papules might have been caused by Dengue infection, suggesting a possible immunological link between viral infection and inflammatory skin disorders. Viral infection might modify self‐antigens on the surface of basal keratinocytes and induce immune dysregulation. Thus, the overactivation of cytotoxic CD8+ cells and uninhibited production of proinflammatory cytokines such as IL‐2, TNF‐α, and IFN‐γ by CD4+ T cells play an important role in the implication of Lichen planus [4]. The association between Dengue fever and this dermatological shift emphasizes the role of immune dysregulation in modifying pre‐existing skin conditions.

There is a paucity of literature showing the association of LP with Dengue infection. Hence, further research is needed to explore the underlying mechanisms linking viral infections with immune‐mediated skin diseases.

## 6 Conclusion

This case highlights an unusual presentation of Lichen planus following a dengue infection. Clinicians should remain vigilant for atypical cutaneous manifestations in post‐viral states, as timely recognition and appropriate management can impact patient outcomes.

## Author Contributions

**Sunil Jaiswal:** conceptualization, data curation, formal analysis, writing – original draft, writing – review and editing. **Shraddha Uprety:** conceptualization, formal analysis, supervision, writing – original draft, writing – review and editing. **Pratichya Thapa:** formal analysis, investigation, methodology, supervision, visualization. **Prakriti Lamichhane:** conceptualization, formal analysis, methodology, writing – original draft.

## Ethics Statement

The patient in this manuscript has given written informed consent for the use of their case details (including photographs) for publication.

## Consent

Written informed consent was obtained from the patient for the publication of this case report and accompanying images.

## Conflicts of Interest

The authors declare no conflicts of interest.

## Data Availability

The data that support the findings of this study are openly available on request.
